# *Thiobacillus* as a key player for biofilm formation in oligotrophic groundwaters of the Fennoscandian Shield

**DOI:** 10.1038/s41522-023-00408-1

**Published:** 2023-06-22

**Authors:** Margarita Lopez-Fernandez, George Westmeijer, Stephanie Turner, Elias Broman, Magnus Ståhle, Stefan Bertilsson, Mark Dopson

**Affiliations:** 1 Centre for Ecology and Evolution in Microbial Model Systems (EEMiS), Linnaeus University, Stuvaregatan 4, 392 31 Kalmar, Sweden; 2 Department of Aquatic Sciences and Assessment, Swedish University of Agricultural Sciences, Box 7050, SE75007 Uppsala, Sweden; 3 Present Address: Department of Microbiology, Faculty of Sciences, University of Granada, Avenida Fuentenueva s/n, 18071 Granada, Spain; 4 Present Address: Department of Forest Mycology and Plant Pathology, Swedish University of Agricultural Sciences, Box 7026, 75007 Uppsala, Sweden; 5 Present Address: Department of Ecology, Environment and Plant Sciences, Stockholm University, Svante Arrhenius väg 20 A, 106 91 Stockholm, Sweden

**Keywords:** Biofilms, Microbial communities

## Abstract

Biofilm formation is a common adaptation for microbes in energy-limited conditions such as those prevalent in the vast deep terrestrial biosphere. However, due to the low biomass and the inaccessible nature of subsurface groundwaters, the microbial populations and genes involved in its formation are understudied. Here, a flow-cell system was designed to investigate biofilm formation under in situ conditions in two groundwaters of contrasting age and geochemistry at the Äspö Hard Rock Laboratory, Sweden. Metatranscriptomes showed *Thiobacillus*, *Sideroxydans*, and *Desulforegula* to be abundant and together accounted for 31% of the transcripts in the biofilm communities. Differential expression analysis highlighted *Thiobacillus* to have a principal role in biofilm formation in these oligotrophic groundwaters by being involved in relevant processes such as the formation of extracellular matrix, quorum sensing, and cell motility. The findings revealed an active biofilm community with sulfur cycling as a prominent mode of energy conservation in the deep biosphere.

## Introduction

The deep biosphere is separated from the photosynthesis-fueled surface both by physical distance and the time that subsurface life has been isolated from aboveground influences. These environments include water-filled fractures in terrestrial bedrock, marine sediments deeper than one meter below the bottom of the sea, and lakes beneath glaciers. The continental deep biosphere extends to several kilometers below the land surface^1^ and is estimated to contain 2–6 × 10^29^ microbial cells^2^ from all three domains of life^3^ and also features viruses^4^. Despite the extremely carbon- and energy-poor conditions prevailing in many deep subsurface environments^5^, life in this biome is key for maintaining the Earth’s biogeochemical cycles^6–9^. Existing data on subsurface populations include metatranscriptomic identification of RNA transcripts^3,10^; a viability study suggesting most taxa have intact cells^11^; and the presence of RNA transcripts assigned to phage particles suggesting that their host microbes are able to reproduce^4^. Sampling the terrestrial deep biosphere requires boreholes, underground laboratories, or deep mines. Consequently, this environment is little explored and contains many novel species for which questions remain as to how they survive in these harsh conditions for up to millions of years and how they may influence global nutrient and energy cycles.

Biofilms are assemblages of microorganisms embedded in a matrix of extracellular polymeric substances (EPS; reviewed in Flemming and Wuertz^12^). These microbes have a set of prominent communal properties that to some degree distinguish them from planktonic cells, including a well-developed ability to cope with fluctuating environmental conditions. Biofilm formation involves both swimming and gliding motility via, e.g., flagella; initial attachment to a conditioned surface involving pili; stabilization of the biofilm by the buildup of a biofilm matrix and then the development of microcolonies that includes the production of EPS; and finally, biofilm maturation (reviewed in Maric and Vrane^13^). Biofilm formation is controlled by regulatory systems including quorum sensing via, e.g., acyl-homoserine lactones (HSLs) and cyclic di-GMP (c-di-GMP) levels. Studies investigating this mode of life in the terrestrial deep biosphere include active bacterial and archaeal biofilms in the Iberian Pyrite Belt^14^, the identification of biofilm cell densities 100-fold greater than the planktonic community^15^, and observations that the minerals on which biofilms form can constrain cell densities and influence community composition^16^. Earlier investigations of biofilm formation in the deep biosphere, using flow-cells connected to groundwaters, showed a high abundance of the sulfate reducers *Desulfovibrio*, *Desulforhopalus*, *Desulfomicrobium*, and *Desulfobulbus*^17^. Additionally, the addition of exogenous sulfate selected for sulfate reducers, while the biofilm communities were overall very similar to the planktonic community^18^. However, more recent genome-resolved metagenomics on biofilm formation contrasted with the studies described above in that the biofilm and planktonic populations were dissimilar and that biofilm formation was largely mediated by populations being capable of oxidizing hydrogen and fixing carbon dioxide and molecular nitrogen to sustain growth^19^. The distinction between surface attached and suspended populations is supported by data from a site at 1450 m depth in South Dakota, USA^20^, and in sedimentary and granitic rock types in Japan^21^. Despite that a substantial fraction of microbial biomass is embedded in biofilms^12^, little attention has thus far been paid to this type of growth in the terrestrial deep biosphere.

The Äspö Hard Rock Laboratory (HRL), operated by the Swedish Nuclear Fuel and Waste Management Company (SKB), is a 3.6 km long tunnel that extends 460 m below sea level on the Baltic Sea coast, southern Sweden. The bedrock is part of the Fennoscandian Shield that consists of 1.8 billion years old Paleoproterozoic granitoids that are crossed by mostly vertical to sub-vertical fractures containing waters of different ages and origins. The geology and hydrology of the site have been previously described^22–24^. In general, the availability of a range of organic carbon sources supports a greater diversity of organisms at shallower depths and the community is primarily dependent on organic carbon infiltration^3,10,25–27^. Below the groundwaters influenced by organic carbon from the surface, microorganisms are suggested to be fueled by hydrogen and carbon dioxide of geological origin^27^. The extremely low availability and recalcitrant nature of organic carbon in these groundwaters^28^ are illustrated by the similarity between total cell numbers and cells with an intact membrane that suggests the microbial community is adapted to the prevailing conditions and rapidly recycles biomolecules such as DNA from dead cells into new biomass^11^. However, active microbial populations and their cellular processes have not been investigated in biofilms from the Fennoscandian Shield deep biosphere.

In the present study, biofilm formation in the deep biosphere was investigated by using a novel flow-cell system attached under in situ temperature and water pressure to two boreholes containing groundwater of contrasting geochemical characteristics. The objectives were to determine (1) which microbial populations comprised the biofilm community and, (2) which clades contributed to the initiation and development of the biofilm. Earlier work has shown that the terrestrial deep biosphere hosts microbial and fungal populations potentially able to form biofilms^19,29^ and that biofilm formation has several stages during which the community may change. Hence, microbial communities were characterized at 20, 40, and 75 days of incubation, using a combination of 16S rRNA gene amplicons and metatranscriptomes. We hypothesize that the initiation of the biofilm community is comprised of specialized clades and therefore has a lower diversity compared to the planktonic community.

## Results and discussion

**Fig. 1:**
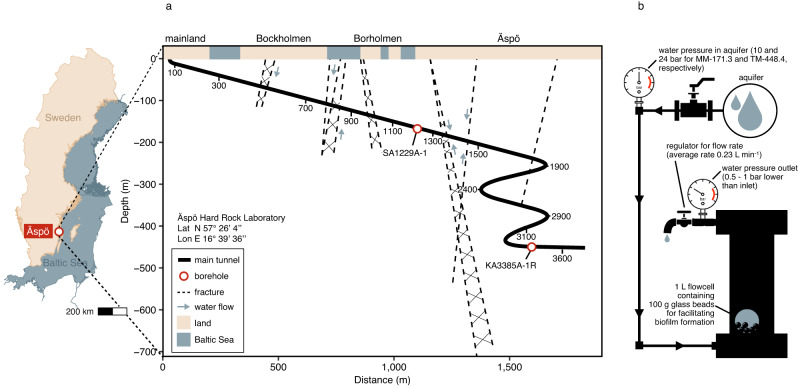
Flow-cell system used for biofilm development. **a** Location of Äspö Hard Rock Laboratory within Sweden with a diagram of the tunnel showing the sampled boreholes and fractures. Figure modified from Pedersen et al.^66^. **b** An illustration of the flow-cell with the solid support and the water flow. The flow-cells were connected to the boreholes under in situ water pressure and sampled in biological duplicates after 20, 40, and 75 days of incubation, resulting in six samples for each of the two groundwater types. After each incubation period, the beads were harvested and subjected to a DNA/RNA co-extraction, and the flow-cell was sterilized and filled with sterile glass beads. The experiment ran between May 2016 and March 2017.

To test if biofilm formation could be established on natural Fennoscandian Shield diorite extracted from the Äspö HRL, crushed rock was added to the flow-cells (Fig. 1). After allowing the biofilm to form, it was not possible to extract biomolecules, probably due to minerals interacting with the nucleic acids producing secondary precipitates that prevented the extraction process. Therefore, glass beads were used as an alternative support for biofilm development (as previously reported^19^) and the extraction was optimized for these samples (Table 1).

**Table 1:** Amplicon and metatranscriptomic sequencing output.

Type	Source	Borehole	Incubation period (days)	*n*	Sampling date	DNA or RNA conc. (ng μL^−1^, mean ± sd)	No. ASVs (DNA) or ORFs (RNA), mean ± sd
DNA	Planktonic	MM-171.3		6	Mar 2016 + Apr 2017	3.2 ± 3.4	1045 ± 660
		TM-448.4		6	Mar 2016 + Apr 2017	0.53 ± 2.3	304 ± 148
	Biofilm	MM-171.3	20	2	May 2016–Mar 2017	0.09 ± 0.05	156 ± 12.7
			40	2	May 2016–Mar 2017	0.16 ± 0.06	290 ± 333
			75	2	Mar 2017	0.14 ± 0.02	39 ± 9.9
		TM-448.4	20	2	May 2016–Mar 2017	<0.05	46 ± 1.4
			40	2	May 2016–Mar 2017	0.16 ± 0.08	129 ± 4.2
			75	2	Mar 2017	0.08 ± 0.02	123 ± 127
RNA	Planktonic	MM-171.3		2	Sep 2015	11.9 ± 7.6	42,063 ± 31,379
		TM-448.4		2	Dec 2015	5.0 ± 1.4	3187 ± 3693
	Biofilm	MM-171.3	75	2	Mar 2017	44.5 ± 2.6*	5310 ± 164
		TM-448.4	75	2	Mar 2017	38.4 ± 11.6*	3993 ± 365
DNA+RNA	Control	MM-171.3		1	Mar 2017	<0.05	
		TM-448.4		1	Mar 2017	<0.05	

In total, the eight metatranscriptomes yielded 84,725 coding regions of which 35% were annotated. The four metatranscriptomes from the planktonic samples were previously published^10^. Sequencing of the 16S rRNA gene amplicons yielded a total of 4682 ASVs. The controls (blank filter collected in parallel to sampling) were subjected to a DNA/RNA co-extraction but neither the amplification generated a product (DNA), nor did the generation of a cDNA library (RNA) work due to insufficient amounts of nucleic acids.

*Showing cDNA concentration instead of RNA concentration as the latter was below the detection limit of the measuring instrument (i.e., 0.05 ng μL^−^1).

*ORF* open reading frame.

### Sampling site and geochemistry

The Äspö HRL (Fig. 1; Lat 57° 26′ 4′′ Lon 16° 39′ 36′′) is an underground tunnel excavated in ~1.8 billion years old granitoids and contains boreholes intersecting fractures of contrasting depth and geochemical characteristics^22–24^. The contamination risk is minimized as these groundwaters have been continually flowing toward the tunnel by hydrostatic pressure since its construction in the 1990s. The geochemistry data included in this study (Table 2) was part of SKB’s monitoring program and shows stability over time^23,27^. The MM-171.3 borehole (SKB reference SA1229A-1) intersects a fracture at 171.3 m below sea level containing groundwater that is characterized by a chloride concentration (87.5 mM) and a δ^18^O value (−7.30‰, relative to Standard Mean Ocean Water) similar to those in the Baltic Sea^23^, revealing recharge from the overlying brackish water^22^. The residence time of this groundwater was estimated to be less than 20 years, containing 0.53 mM of dissolved organic carbon (DOC) and 4.17 mM carbonate and therefore, this fissure was defined as a ‘modern marine’ groundwater. The TM-448.4 borehole (SKB reference KA3385A-1R) intersects groundwater at 448.4 m below sea level and had a chloride concentration (211.3 mM) and an intermediate δ^18^O ratio (−10.8‰) between that of the modern marine and the ancient, more saline groundwaters^27^. This fissure had a lower concentration of DOC (0.11 mM) and carbonate (0.352 mM) than the MM-171.3 groundwater. Hence, this fissure was referred to as a ‘thoroughly mixed’ groundwater as it was composed of unknown proportions of different water types, and therefore its age could not be accurately determined. The relatively high concentration of sulfate in both groundwaters (0.94 mM and 1.5 mM for MM-171.3 and TM-448.4, respectively) suggested this could be a valuable electron acceptor for sulfate-reducing bacteria. However, previous studies on the terrestrial deep biosphere demonstrate active sulfur cycling^5,8^ while labile organic matter appears to be scarce^28^, illustrating microbial activity that would not be detected by geochemical analysis alone.

**Table 2:** Groundwater chemistry.

	MM-171.3	TM-448.4
Depth (m)	171.26	448.35
pH	7.3	7.5
EC (mS cm^−1^)	1002	2093
δ^18^O (‰)	−7.3	−10.8
DOC	0.53	0.11
HCO_3_^−^	4.17	0.352
NH_4_^+^	0.281	3.33e^−3^
NO_3_^−^	BD	1.6e^−5^
NO_2_^−^	1.1e^−5^	4.3e^−6^
Fe^2+^	3.22e^−2^	1.59e^−2^
Mg^2+^	5.76	2.39
SO_4_^2^^−^	0.94	1.47
HS^−^	2.18e^−3^	3.12e^−4^
Cl^−^	87.53	211.3

Values are concentrations in mM unless otherwise stated. δ^18^O (‰) is the ^18^O/^16^O ratio relative to Standard Mean Ocean Water. Nitrate concentration of the MM-171.3 was below detection limit (BD; <4.8e^−6^ mM).

*DOC* dissolved organic carbon, *EC* electrical conductivity, *BD* below detection limit.

### Amplicon and metatranscriptomic sequencing output

The biofilm communities were characterized as biological duplicates after 20, 40, and 75 days of incubation in two independent flow-cells for each of the MM-171.3 and TM-448.4 water types (*n* = 6 for each groundwater, Table 1). The planktonic cells were captured before (*n* = 3 for each groundwater type) and after (*n* = 3) the period of biofilm formation in the flow-cells to ensure the planktonic communities were fully characterized. The 16S rRNA gene amplicon samples (*n* = 24) generated on average 70,003 reads (range 2194–209,741) and yielded a total of 4682 amplicon sequence variants (ASVs) of which 85% and 66% were characterized at the level of order and genus, respectively. Rarefaction curves for both planktonic and biofilm samples were asymptotic (Supplementary Fig. 2), suggesting the low number of ASVs in the biofilm was a biological phenomenon related to a limited number of founder populations during early stages of biofilm development^19^.

Metatranscriptomes from 75-day-old biofilms (*n* = 2 for each groundwater) were compared with published planktonic metatranscriptomes from the same groundwaters (*n* = 2 for each groundwater)^10^. While the planktonic and biofilm metatranscriptomes were collected at different times, a study of metatranscriptomes from identical groundwaters sampled several years apart showed stable planktonic communities over time^10^. Attempts were made to generate metatranscriptomes from 20- and 40-day-old biofilms and despite cDNA synthesis, only RNA from 75-day-old biofilms could be successfully sequenced. This was likely due to limited biomass during early-stage biofilm formation in low-energy environments such as the groundwaters under scrutiny here. In total, all eight metatranscriptomes produced an average of 42.1 million reads per sample (range 32.4–53.4 million) and contained a total of 84,725 unique open reading frames of which 35% and 38% were taxonomically and functionally annotated, respectively (Table 1).

The genera *Ralstonia* (Burkholderiales), *Brevundimonas* (Caulobacterales), and *Hoeflea* (Hyphomicrobiales) have been described as notorious kit contaminants^30,31^ and were present in the amplicon dataset. Despite attempting parallel amplification of extraction controls, no product of sufficient concentration to allow sequencing was obtained. *Ralstonia* and *Brevundimonas* were also present in the metatranscriptomes of the biofilm communities, albeit in low abundance (0.75 ± 0.35%, *n* = 4), indicating that they only played a minor role or were less active during biofilm formation. As sequencing of the extraction controls failed, it cannot be ruled out that these populations were contaminants introduced during the molecular work. However, as populations assigned to these genera only played a marginal role in biofilm formation, this did not alter the interpretation of the data.

### DNA-based biofilm communities

**Fig. 2:**
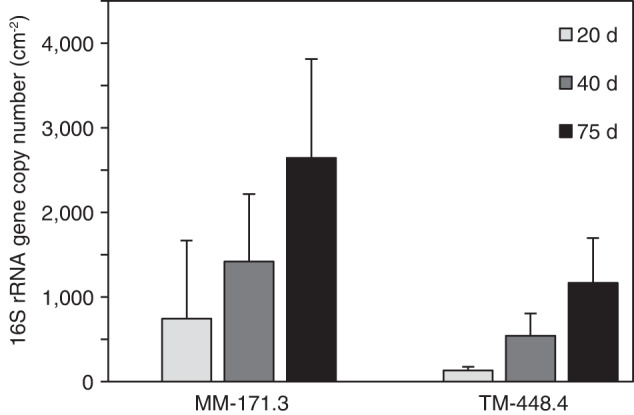
Bacterial abundances for biofilm samples based on qPCR. Bar plot showing the bacterial 16S rRNA gene copy numbers per surface area (cm^−2^) of the glass beads in the flow-cells after 20, 40, or 75 days of biofilm formation for both groundwater types (MM-171.3 and TM-448.4). Archaeal abundances were below detection limit and were therefore not included. Error bars denote the standard deviation among the replicates (*n* = 2).

Quantification of 16S rRNA genes via quantitative real-time PCR (qPCR) in the biofilm samples revealed an increased bacterial abundance with incubation time for both water types (nested ANOVA, F = 5.4, *p* = 0.03; Fig. 2), whereas archaeal abundances were consistently below detection limit. Bacterial abundances were higher in MM-171.3 (747–2647 gene copies cm^−2^) as compared to TM-448.4 biofilms (134–1168 gene copies cm^−2^) potentially related to the higher content of organic carbon in this groundwater. Compared to similar experiments, the bacterial abundances in this study were toward the lower end of values reported in the literature (216 to 400,000 gene copies cm^−2^) for Äspö HRL biofilm communities^17,32^. This was probably due to the short incubation time in this study and also depends on the overall characteristics of the groundwater.

**Fig. 3:**
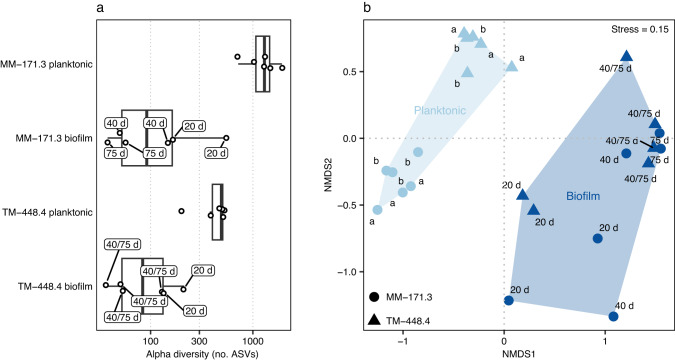
Diversity based on 16S rRNA gene amplicons. **a** Boxplot combined with a dot plot depicting alpha diversity of both biofilm (*n* = 12) and planktonic (*n* = 12) communities, according to the number of ASVs within each sample. Labels on the biofilm samples denote the incubation period in the flow-cell. The box is formed by the first and third quartiles with the whiskers extending to 1.5 times the interquartile range. **b** Beta diversity according to an NMDS on Bray–Curtis dissimilarities. Labels on the biofilm samples denote the incubation period to allow biofilm formation in the flow-cell of either 20, 40, or 75 days. Labels on the planktonic samples denote the sampling date with ‘b’ for before the incubation experiment (March 2016) and ‘a’ for after the experiment (April 2017).

**Fig. 4:**
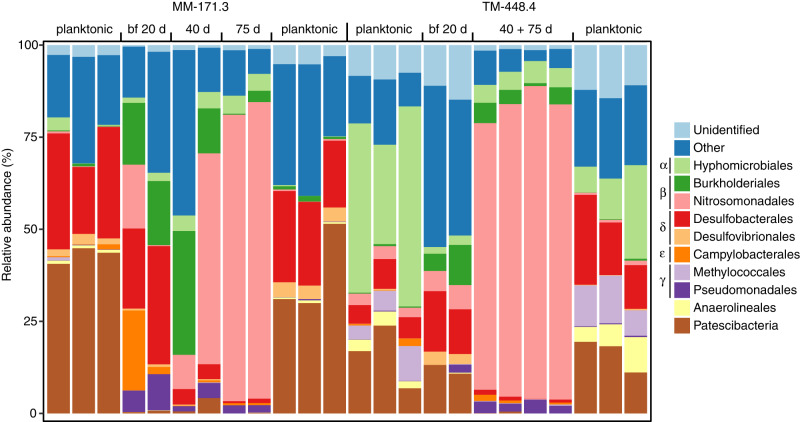
Microbial community structure based on gene amplicons. Composition of both biofilm (bf) and planktonic communities, sorted at the taxonomic level of order. The planktonic community of each groundwater was characterized (*n* = 3) before (March 2016) and after (April 2017) the incubation period of the flow-cells to verify the community composition had not shifted during the course of running the experiment. The ten most abundant orders are shown with the remaining taxa grouped as “Other”. ASVs not identified on the level of order are grouped as “Unidentified”. Greek letters before the color key denote the class within the Proteobacteria (Alpha, Beta, Delta, Epsilon, and Gammaproteobacteria). The Patescibacteria are shown as phylum as the individual orders are mainly placeholder names.

Characterization of the microbial communities using 16S rRNA gene amplicons yielded a total of 964 and 4063 ASVs for the biofilm and planktonic communities, respectively. Alpha diversity was lower in the biofilms compared to the planktonic communities (nested ANOVA, F = 46, *p* = 2.3e^−3^, Fig. 3). Additionally, the biofilm communities (*n* = 12) featured fewer phyla (40), orders (184), and genera (386) compared to the planktonic communities (*n* = 12) that comprised 74 phyla, 327 orders, and 721 genera. Furthermore, alpha diversity for both biofilm communities decreased over time with the diversity at 20 days being the highest (Fig. 3), followed by a decrease after 40 and 75 days of incubation. Beta diversity analysis revealed a change between biofilm and planktonic communities (PERMANOVA, *R*^2^ = 0.24, *p* = 0.001; Fig. 3), confirming that deep biosphere biofilm communities were distinct from their respective planktonic communities^19^. The clade mainly responsible for this change was the genus *Thiobacillus* (Nitrosomonadales) that dominated the biofilm communities of both groundwater types after 75 days of incubation (relative abundance > 50%; Fig. 4 and Supplementary Fig. 3). The involvement of *Thiobacillus* in the biofilm communities from both groundwaters was also apparent from the differential abundance analysis with five out of ten most differentially abundant ASVs (Welch’s *t*-test, *p* < 0.05) affiliated with this genus, along with *Desulfomicrobium* (Desulfobacterales), *Sulfurimonas* (Campylobacterales), *Pseudomonas* (Pseudomonadales), and *Methylotenera* (Nitrosomonadales). *Thiobacillus* populations were previously identified in the biofilm community of identical groundwaters^19^, and are described as aquatic biofilm formers^33^ that can dominate biofilms together with *Sulfurimonas*^34^. Like *Thiobacillus*, *Sulfurimonas* is known for oxidizing sulfur coupled to nitrate reduction and is involved in surface attachment to enable biofilm formation in various ecosystems^25,33–36^. *Pseudomonas aeruginosa* is a model organism for biofilm development due to its secretion of biofilm matrix compounds, contributing to biofilm formation and maturation under a wide range of conditions^37^. Finally, *Methylotenera* also secretes extracellular polymers as part of biofilm initiation^38^. In the early stage of biofilm formation (20 days incubation) the sulfate-reducing orders Desulfobacterales and Desulfovibrionales were abundant while in later stages (40 and 75 days) these clades were rare. A similar pattern was observed for the Spirochetes and Patescibacteria that were abundant in the early-stage TM-448.4 biofilms while the abundance of these phyla declined over time as the biofilm matured (Supplementary Fig. 3). Patescibacteria have mainly been described in groundwater systems and are suggested symbionts. They typically have small genomes and often lack many biosynthetic pathways. Based on these observations, representatives from this phylum may lack the metabolic capacity for biofilm formation and therefore are unable to produce (or fail to attach to) an extracellular matrix. Finally, the observed change in community structure between the early stage (20 days) and later stages (40 and 75 days) of biofilm formation suggested a biofilm community in development was characterized by pronounced changes in composition over time.

Comparing the amplicon-based biofilm communities with those from other studies on biofilms in the subsurface showed a common high abundance of Alpha-, Beta-, and Gammaproteobacteria^16,20,29,39^. *Thiobacillus* is described as a prominent community member in sulfidic cave biofilms in Italy^40,41^ and was described as abundant during the later stages of community succession in a biofilm reactor enriching for sulfur-oxidizing denitrifiers^42^. However, in most studies, comparisons at higher taxonomic resolution revealed differences among the attached bacterial communities. For instance, neither Desulfobacterales nor Nitrosomonadales was identified as abundant clades in the bacterial biofilm community in a study on the continental deep biosphere at Outokumpu, Finland^29^ or found in a former gold mine in the USA^20^, nor in a study on mineral-hosted biofilm communities in the Deep Mine Microbial Observatory, USA^16^. Interestingly, two out of four of the before mentioned studies^20,39^ also reported Hyphomicrobiales to be abundant in the biofilm community, even though representatives of this clade are usually associated with plant roots by the fixation of atmospheric nitrogen^43^. In general, the high variation among the attached bacterial communities illustrated the influence of local factors in shaping microbial communities, such as host rock or mineral^16^, electron donor and/or acceptor^20^, fungal-bacterial interactions^29^, or the whether the biofilm was from a terrestrial or marine environment^39^.

According to the beta diversity analysis, there was a clear differentiation among planktonic and biofilm communities and this change was mainly driven by the dominance of *Thiobacillus*, *Methylotenera*, and *Pseudomonas* during the later stages of biofilm formation (Fig. 3). Diversity analysis, community composition, and especially the dominance of a limited number of genera showed that a specialized community was involved in the biofilm formation. Finally, the low abundance of the biofilm community in general and the strong increase of bacterial abundance over time (20, 40, and 75 days of incubation) showed that the observed microbial communities were indeed from a developing biofilm.

### RNA-based biofilm communities

**Fig. 5:**
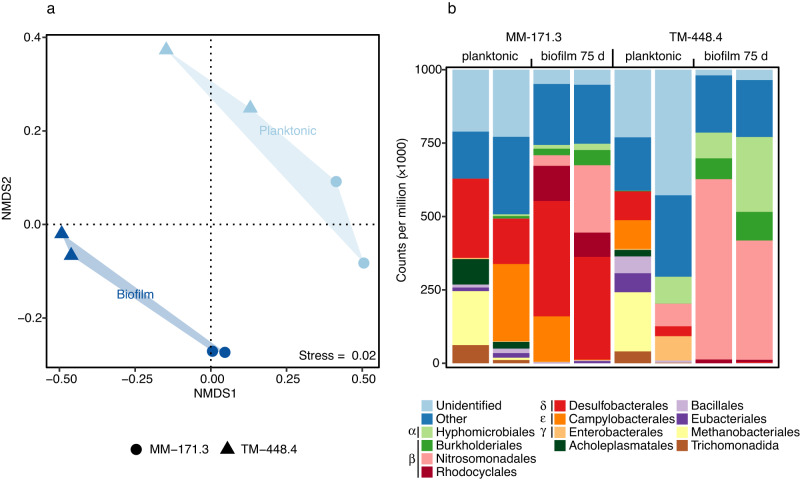
Microbial community structure based on RNA transcripts. **a** Beta diversity according to an NMDS on Bray–Curtis dissimilarities for mRNA transcripts from the planktonic and 75 days old biofilm communities and **b** composition of planktonic and 75 days old biofilm communities, sorted at the taxonomic level of order. The 12 most abundant orders are shown with the remaining taxa grouped as “Other” and transcripts not annotated on the level of order grouped as “Unidentified”. Greek letters before the color code denote the class within the Proteobacteria (Alpha, Beta, Delta, Epsilon, and Gammaproteobacteria); 13.2 and 22.6% of these unidentified transcripts for the MM-171.3 and TM-448.4 groundwaters, respectively, were Bacteria without any further classification on a higher taxonomic resolution. Data for the planktonic RNA transcripts are from Lopez-Fernandez et al.^10^.

In total, 4837 and 3250 annotated transcripts were identified in the metatranscriptomes of the MM-171.3 and TM-448.4 biofilm communities, respectively, compared to 23,831 and 2706 annotated transcripts in their planktonic counterparts. The RNA-based activity in the biofilm community was different from that in the planktonic community (PERMANOVA, *R*^2^ = 0.25, *p* = 0.032) and showed a high variation among the two groundwater types (Fig. 5). This variation among the biofilm communities was mainly due to the high abundance of *Thiobacillus denitrificans* (Nitrosomonadales) in the TM-448.4 biofilm while the most abundant genera in the MM-171.3 biofilm were *Desulforegula* (Desulfobacterales), *Sideroxydans* (Nitrosomonadales), and *Dechloromonas* (Rhodocyclales). These four genera have all been described in deep biosphere groundwaters as being both abundant and active in either the oxidation of reduced sulfur compounds (*Thiobacillus, Sideroxydans*, and *Dechloromonas*) or involved in sulfate reduction (*Desulforegula*)^5,8^. In general, the MM-171.3 biofilm contained more active clades (72 unique orders) compared to the TM-448.4 biofilm (56 orders), possibly due to the former groundwater being more connected to surface waters and having a higher organic carbon content (Table 2). Similar to what was observed in the amplicon data, the active biofilm communities contained fewer taxa at the level of phylum (19), order (57), and genus (66) compared to the planktonic community (27, 72, and 87, respectively). These results are in line with previous studies on biofilm formation located in Äspö HRL^19^ and a former gold mine in Wyoming, USA^20^, that proposed a model where a subset of the planktonic microbial community attaches to the solid surface and initiates biofilm formation. Comparing the composition of the biofilm communities based on 16S amplicons (Fig. 4) with the communities based on RNA transcripts (Fig. 5) revealed that Desulfobacterales were only marginally present in the MM-171.3 amplicon biofilm samples while being the most abundant group in the biofilm transcriptomes from this groundwater. A similar pattern was observed for the Hyphomicrobiales in the TM-448.4 biofilm transcriptomes. This discrepancy could have several causes, such as a primer bias for certain amplicon variants, a better annotation of the transcripts for particular taxonomic groups, or low transcriptional activity of the Nitrosomonadales in the MM-171.3 biofilm despite a high abundance. In contrast, the Patescibacteria such as *Candidatus* Falkowbacteria were abundant in the amplicon dataset (Supplementary Fig. 3) yet were nearly absent in the transcriptomes. This was likely because Patescibacteria are proposed to be episymbionts and scavengers that do not contribute to the common goods^44^ and that there was a selection for microorganisms responsible for the energy metabolism necessary for the biofilm formation.

Eukaryotes were more abundant in the planktonic communities compared to the biofilms, with 8.7% and 8.2% of the annotated transcripts affiliated with this group (1.0% and 0.2% in the biofilms) in the MM-171.3 and TM-448.4 groundwaters, respectively. These transcripts could rarely be identified at the level of order and therefore are not depicted as a separate group in Fig. 5 and were included as ‘Unidentified’ taxa. Active eukaryotes have previously been identified in groundwaters at Äspö HRL^3,10^ and other continental deep subsurface sites^29,45^. As fungi play a role in deep biosphere biofilms^19,29^, the metatranscriptomes were interrogated for fungal transcripts that revealed 0.52% and 0.15% of the annotated transcripts for the MM-171.3 and TM-448.4 biofilms, respectively (Supplementary Fig. 4). A reason for the poor annotation of the eukaryotic transcripts could be that the software used for annotation (Prokka) was optimized for annotating bacterial, archaeal, and viral genomes^46^. However, the data suggested that eukaryotes played a minor role in the biofilm formation.

### Biofilm formation

**Fig. 6:**
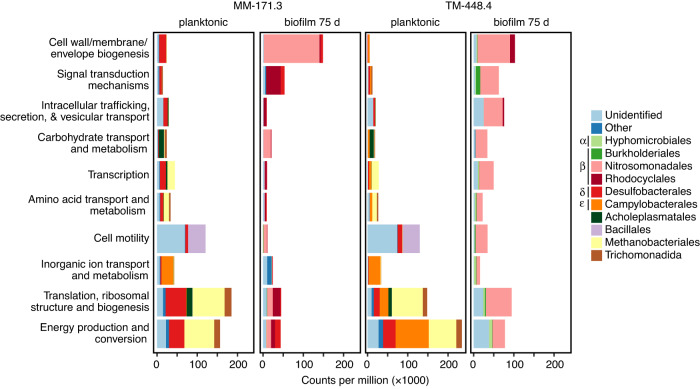
Differentially expressed RNA transcripts grouped by functional category. Transcripts shown are differentially expressed (false discovery rate < 0.05) between biofilm and planktonic communities of the respective groundwater types. As only two categories were used for the differential expression analysis, a gene being upregulated in the biofilm community implies it was downregulated in the planktonic community and vice versa. The ten most abundant orders are shown with the remaining taxa grouped as “Other” and transcripts not annotated on the level of order grouped as “Unidentified”. Greek letters before the color code denote the class within the Proteobacteria (Alpha, Beta, Delta, and Epsilonproteobacteria). A cluster of orthologous genes (COG) was used to group the individual genes according to function^10^.

Out of the 7941 unique RNA transcripts included in the differential expression analysis, 704 were upregulated (i.e., having higher transcript numbers) in biofilm communities within the context of differential expression analysis (false discovery rate < 0.05), and 1472 were upregulated in planktonic communities. Of the total transcripts, 38% were functionally annotated using the eggNOG-mapper. Results from this analysis represented changes in transcript numbers between the planktonic and biofilm communities, irrespective of the groundwater type (Fig. 6).

*Thiobacillus* populations were responsible for 36% of all upregulated transcripts in the biofilm communities, including genes encoding sulfur oxidation (*fccB*) coupled to dissimilatory nitrate reduction (*napA*). This contribution was likely an underestimation as only 54% of the transcripts with a functional annotation were characterized at the level of genus. Earlier studies demonstrate a primary role of *Thiobacillus* in sulfur-driven denitrification in either deep biosphere aquifers^5^ or in a biofilm reactor^42^. The geochemistry of the groundwaters in this study suggested the electron donor to be limiting, i.e., the concentration of reduced sulfur (<5 µM) was considerably lower than the sulfate concentration (0.94 mM and 1.47 mM for MM-171.3 and TM-448.4, respectively; Table 2). A possible source of reduced sulfur could be from cryptic sulfur cycling^8^, using the end product from sulfate reducers such as Desulfobacteraceae or Desulfobulbaceae (both Desulfobacterales)^5^ as previously described for the Fennoscandian Shield^8^. The high abundance of transcripts coding for genes involved in sulfur reduction supported the presence of such processes (Supplementary Fig. 5) although the Desulfobacterales or other clades performing sulfate reduction were only scarcely present in the TM-448.4 biofilm community (Fig. 5). An explanation for this discrepancy could be that the majority of the transcripts affiliated with sulfate reduction were only identified at the level of phylum as Proteobacteria.

Almost half (45%) of all upregulated biofilm transcripts in the functional group of ‘cell wall/membrane/envelope biogenesis’ were assigned to *Thiobacillus*, this functional group being central in biofilm production^12^. These transcripts included genes coding for flagella proteins (e.g., *flgAE* and *flhA*), twitching and gliding mobility (*pilT* and *frzE*), and alginate biosynthesis (*algA*). *Thiobacillus* was responsible for 53% of all upregulated transcripts categorized as ‘intracellular trafficking, secretion, and vesicular transport’ with transcripts comprising this category (e.g., *epsF* and *exbD*) being involved in extracellular matrix protein transport^47^. Signal transduction was also upregulated in the biofilm, mainly by *Thiobacillus* and *Dechloromonas* (Rhodocyclales) in the MM-171.3 and TM-448.4 communities, respectively. Genes from this category (*rpfG*, *cheAW*, and *pleD*) are involved in quorum sensing and chemotaxis and mediate the transition from a planktonic to a biofilm habitat.

The methanogen *Methanobacterium* (Methanobacteriales) plus the bacterial clades Desulfobacteraceae (Desulfobacterales) and *Sulfurimonas* (Campylobacterales) were affiliated with the majority of transcripts in the functional groups ‘energy production and conversion’ and ‘translation, ribosomal structure, and biogenesis’ (Supplementary Fig. 6). *Methanobacterium* is capable of using hydrogen to reduce either carbon dioxide or formate and potentially has a key role in the planktonic communities due to its autotrophic lifestyle^48^. Together with *Methanobacterium*, the Desulfobacteraceae and *Sulfurimonas* have previously been described in fractures of the Fennoscandian Shield^8,48^, of which the latter two clades were also active in the biofilm communities (Supplementary Fig. 6), yet many of these transcripts were also detected in the planktonic communities and were therefore not flagged as being differentially expressed. *Methanobacterium* had only marginal activity in the biofilm communities, and this was potentially due to a restricted gas flux of, for example, methane in the biofilm matrix. However, transcripts such as *tktA, cmpR*, and *fbp*, involved in the fixation of carbon dioxide as part of the Calvin cycle, were upregulated in the biofilm communities that support previous metagenomic studies on biofilm formation in Äspö HRL aquifers^19^. Stress-related genes such as cold shock genes (*csp* family), cold shock-like genes (*cspL*), and chaperone proteins (*dnaJK*, *clpB*, *groLS*, *htpG*, *dmsD*, *surA*, and *torD*) were mainly categorized as ‘posttranslational modification, protein turnover, chaperones’ and were all downregulated in the biofilm communities. Fewer chaperones in the attached communities also supported the ability of biofilms to protect cells from stress conditions^12^. Finally, the higher abundance of MM-171.3 biofilm transcripts within ‘energy production and conversion’ compared to the TM-448.4 biofilm (Fig. 6) was most likely due to the input of labile organic carbon from the surface in the former groundwater^28^, thereby possibly sustaining a more metabolically active community. This supported previous studies wherein the TM-448.4 groundwater community was described as being in a ‘metabolic standby’ due to the scarcity of energy and nutrients^3^.

This study characterized biofilm communities in deep biosphere groundwaters after 20, 40, and 75 days of incubation in flow-cells. 16S rRNA gene amplicons and metatranscriptomes revealed a biofilm community distinct from its planktonic source, both in terms of beta diversity and with fewer taxonomic groups in biofilm communities at the level of phylum, order, and genus. Comparing the biofilm communities from both groundwater types showed that the MM-171.3 biofilm with the groundwater having a higher organic carbon content and being more connected to resource inputs from the surface water featured more unique annotated transcripts (4837 versus 3250), and hosted a higher number of taxonomic groups (72 versus 56 orders). *Thiobacillus* (Nitrosomonadales) was identified as a key player in biofilm formation, especially in the TM-448.4 groundwater, not only due to its abundance but primarily due to many upregulated transcripts involved in processes such as quorum sensing, cell motility and attachment, and formation of extracellular polymeric substances. Especially for the MM-171.3 biofilm, RNA transcripts for sulfur oxidation and reduction implied cryptic sulfur cycling as a prominent mode of energy conservation. Finally, 75 days of incubation appeared to capture mainly early biofilm formation and future studies on the deep biosphere could extend this incubation period to go beyond this initial biofilm establishment phase.

## Methods

### Planktonic cell capture, DNA extraction, and amplicon sequencing

Planktonic cells were collected in triplicates from the MM-171.3 and TM-448.4 groundwaters before (March 2016) and after (April 2017) biofilm formation experiments in the flow-cell. To avoid contamination from the stagnant water, five section volumes of borehole water were flushed before collecting cells on sterile hydrophilic polyvinylidene fluoride (PVDF) membranes with 0.1 µm pore-size (47 mm Durapore, Merck Millipore) under in situ conditions as previously described^27^. After filtering an appropriate volume of water (Supplementary Table 1), filters were aseptically placed in cryogenic tubes (Thermo Scientific), immediately frozen in liquid nitrogen, transported to the laboratory, and stored at −80 °C until further processing. DNA extraction was performed by using the MO BIO PowerWater DNA isolation kit following the manufacturer’s instructions, except for adjusting the elution volume to 30 µL. The extracted DNA was analyzed by gel electrophoresis and a Qubit 2.0 Fluorometer (Life Technologies) and stored at −20 °C until further processing. The V3-V4 region of the 16S rRNA gene was amplified using the 341F 805R primer pair^49^ according to published procedure^50^. Finally, sequencing was performed at the Science for Life Laboratory, Sweden on an Illumina MiSeq, producing 2 × 301 bp paired-end reads. While the utilized primers 341F and 805R were originally designed to amplify bacterial 16S rRNA gene sequences, they have been demonstrated to amplify part of the archaeal diversity from groundwaters at the Äspö HRL^27^. To address this primer bias, archaeal abundances were also assessed by conducting a qPCR with archaeal primers (described below). Two negative controls were performed by extracting DNA from unused filters collected in parallel to sampling both groundwaters. The extracts of these controls had DNA concentrations below the detection limit of the Qubit 2.0 Fluorometer (i.e., <0.05 ng µL^−1^; Life Technologies). Amplification of these DNA extracts was attempted but no product was detected, neither by using agarose gel electrophoresis nor with the Qubit.

### Flow-cell for biofilm formation

Two flow-cells (Fig. 1 and Supplementary Fig. 1) were constructed using biologically inert materials by Maskinteknik AB, Oskarshamn, Sweden. After flushing five borehole section volumes, the sterile (via washing in absolute ethanol) flow-cell was filled with either 100 g of 6 mm diameter glass beads (VWR) or crushed natural silicate mineral rock (diorite) extracted from Äspö HRL and attached to the borehole. The diorite sample from the Äspö HRL was washed several times and sterilized with absolute ethanol while the glass beads were sterilized by autoclaving. The average flow rate of the groundwater was 0.23 L min^−1^, allowing the biofilm to form on the glass beads’ surface under in situ water pressure. The decrease in water pressure over time ranged from 0.5 to 1 bar. Biofilms were collected from the flow-cells in biological duplicates after 20, 40, and 75 days making a total of six samples for each of the two groundwaters. To do so, one flow-cell was connected to each borehole during the designated incubation period, from which the content was harvested prior to starting up a new incubation period. The flow-cell was cleaned and sterilized using absolute ethanol, and subsequently filled with new beads between each incubation period. To harvest the biofilm, the water flow was stopped, the pressure was released, and the biofilm cells were immediately (<10 s) fixed by transferring the glass beads to a bottle containing 100 mL of a 5% (vol/vol) water-saturated phenol in absolute ethanol stop solution^3,10^. The fixed cells were immediately frozen in liquid nitrogen and transported to the laboratory where they were stored at −80 °C until the next day when they were processed.

### Nucleic acids extraction, quantitative PCR, and sequencing

The samples were defrosted before extracting nucleic acids using the RNA/DNA/Protein Purification Plus Kit (Norgen Biotek). Then, 20 mL of buffer (50 mM sodium acetate pH 5.5, 2 mM ethylenediaminetetraacetic acid (EDTA), and 0.1% sodium dodecyl sulfate (SDS) were added to the samples and vortexed at maximum speed for 10 min. After that, samples were centrifuged for 15 min at 3000×*g* and 4 °C. The supernatant was carefully decanted, and the pellet was thoroughly resuspended in 100 µL of lysozyme-containing (3 mg mL^−1^) TE buffer by vortexing before incubating at room temperature for 10 min. A final step adding 300 µL of lysis buffer Q (from the Norgen Biotek kit) containing 3 µL of β-mercaptoethanol (Thermo Fisher Scientific) and vigorous vortexing for at least 10 s was performed before proceeding to step two of the RNA/DNA/Protein Purification Plus Kit protocol (Norgen Biotek). The quantity and quality of the extracted RNA and DNA were analyzed with a Qubit 2.0 Fluorometer (Life Technologies) and by agarose gel electrophoresis, respectively. To demonstrate that all the cells had been detached from the glass beads, the washing procedure was repeated that yielded no additional detached cells. Amplification and sequencing of the 16S rRNA gene V3-V4 region were performed identically as described above for the planktonic samples. Finally, due to an issue during the library preparation, it was not possible to distinguish the TM-448.4 biofilm samples of 40 and 75 days incubation and these have been combined in Figs. 3 and 4. However, due to the highly similar results for these samples, this does not alter the conclusions drawn from this study.

To determine the number of archaeal and bacterial 16S rRNA gene copies in the DNA biofilm samples, qPCR was performed on a LightCycler® 480 Instrument (Roche Diagnostics). For both assays, the qPCR reactions contained 5 µL Platinum™ SYBR™ Green qPCR SuperMix-UDG with ROX, 0.4 µL of each primer (10 µM), 3.2 µL nuclease-free water, and 1 µL of DNA template (total volume 10 µL per reaction). Archaeal and bacterial 16S rRNA gene fragments were amplified with primers Arch915F/Arch1059R^51^ and Bac908F_mod/Bac1075R^52^, respectively. Cycling conditions were 2 min at 95 °C, 40 cycles of 15 s at 95 °C, and 30 s at 60 °C (fluorescence measurement), followed by a melt curve analysis to check for primer specificity. In addition, product size was confirmed via gel electrophoresis. Standard curves were based on a dilution series of cleaned PCR product amplified from genomic DNA of pure cultures (*Ferroplasma acidiphilum* BRGM4 for archaea and *Acidiphilium cryptum* JF-5 for bacteria). Standards, template DNA, and no-template controls (water) were run in triplicate reactions. Template DNA was run in two dilutions to check for PCR inhibition. Efficiencies for the archaeal and bacterial assay were 94.7% and 95.9%, respectively. The abundances were reported as gene copy numbers per cm^2^ surface area of the glass beads. To test for significant differences in the gene copy numbers during biofilm formation, an ANOVA with incubation period nested within groundwater type was performed.

DNA contamination of the RNA extractions was checked by PCR with 16S rRNA gene-specific primers 341F and 805R^1^. If no PCR products were obtained, the extracted RNA was utilized to generate cDNA using the Ovation^®^ RNA-Seq System V2 (NuGEN) following the manufacturer’s instructions. Afterward, the generated cDNA was purified using the QIAGEN MinElute Reaction Cleanup Kit. The quantity and quality of the generated cDNA were analyzed as described above (Table 1). Two negative controls were included in the sequencing library: an extraction control to identify potential contaminants in the extraction kit and a control for the cDNA synthesis. cDNA library preparation and sequencing were performed at the Science for Life Laboratory, Sweden (www.scilifelab.se). Library preparation was carried out using the Illumina HiSeq TruSeq Nano DNA Library Prep Kit for NeoPrep. Samples were sequenced on HiSeq2500 with a 2 × 126 bp setup using ‘HiSeq SBS Kit v4’ chemistry.

### Bioinformatic analysis of 16S rRNA gene amplicons

Raw sequencing reads were processed with default settings using the Ampliseq pipeline^53^ (v1.2.0) that relied on Nextflow (v21.10.6), FastQC (v0.11.9), Cutadapt (v3.4), MultiQC (v1.9), DADA2 (v1.22.0), and the SBDI Sativa curated 16S GTDB database^54^ (release 207). The number of reads throughout the bioinformatic pipeline and the amount of ASVs are shown in Supplementary Table 1. Absolute sequencing counts were standardized to relative abundances by dividing an ASV’s count by the total number of reads within a sample^55^. Alpha diversity was estimated using the number of ASVs. To test for differences in alpha diversities between water types and biofilm versus planktonic cells, an ANOVA was used with planktonic or biofilm samples nested within groundwater type (R package stats, v3.6.3). Post-hoc testing was done with Tukey’s HSD test using a Bonferroni correction for multiple comparisons. Beta diversity was estimated according to Bray–Curtis dissimilarities and visualized using nonmetric multidimensional scaling (NMDS). Statistical testing of the beta diversity was done by means of a permutational analysis of variance (PERMANOVA), setting the number of permutations to 999, and correcting multiple comparisons with the Bonferroni correction. The R Vegan library^56^ (v2.5-7) was used on default settings for estimating both alpha and beta diversity and for the statistical testing of diversity indices. Differentially abundant taxa between communities were identified by using the R package ALDEx2 (v1.18.0) which combines a log-ratio transformation and statistical testing using the non-parametric Welch’s *t*-test.

### Bioinformatic analysis of RNA transcripts

Illumina adapters were trimmed from the raw reads using Cutadapt (v3.1) on default settings followed by inspection of the trimmed reads using FastQC (v0.11.8) and MultiQC (v1.9). Small subunit (SSU) ribosomal RNA was removed by aligning the trimmed reads to the Silva 138.1 reference database^57^ using BBDuk (v38.61). The retained reads were assembled using Megahit^58^ (v1.2.9) while Prokka^46^ (v1.12) was used for functional annotation. Predicted proteins were categorized using the eggNOG-mapper^59^ (v2.2.1). Diamond^60^ (v2.0.4.) was used for alignment and Megan^61^ (v6.21) was used for taxonomy assignment combined with the NCBI database^62^ (v5). Trimmed reads were mapped to the contigs using Bowtie2^63^ (v2.3.5.1) and the abundance of the open reading frames was quantified with featureCounts^64^ (v2.0.0). The absolute transcript count per sample was standardized as counts per million (cpm) using the R library edgeR^65^ (v3.28.1). This library was also used for differential expression analysis (false discovery rate < 0.05) between the planktonic and biofilm communities. As the 16S rRNA gene amplicons and the RNA transcripts were annotated using different reference databases (GTDB vs. NCBI), this caused minor differences in the taxonomy, especially in the class Gammaproteobacteria. To resolve this, the taxonomy of the NCBI reference database was used for all downstream analyses.

### Reporting summary

Further information on research design is available in the Nature Research Reporting Summary linked to this article.

## Supplementary information

Supplemental material

Reporting Summary

## Data Availability

The datasets generated for this study can be found in the European nucleotide archive under the study reference PRJEB47594.

## Code availability

R markdown files used for data analysis and figure generation plus the functional annotation of the RNA transcripts are available at GitHub https://github.com/geweaa/flowcells.
