# Supplementary material for: Thiobacillus as a key player for biofilm formation in oligotrophic groundwaters of the Fennoscandian Shield
Source: NPJ Biofilms Microbiomes. 2023 Jun 22;9:41. doi: 10.1038/s41522-023-00408-1 (PMC10287647; doi:10.1038/s41522-023-00408-1)
Supplement: Supplementary file 1 — Supplemental material [file 41522_2023_408_MOESM1_ESM.pdf]

Thiobacillus as a key player for biofilm formation in oligotrophic groundwaters of the Fennoscandian Shield

Margarita Lopez-Fernandez, George Westmeijer, Stephanie Turner, Elias Broman, Magnus Ståhle, Stefan Bertilsson, & Mark Dopson

Supplementary Materials

Supplementary Table 1. Sequencing details DNA and RNA. Overview of the sequencing data used in this study and the volume of groundwater filtered prior to the molecular work.

	Sample	Type	Groundwater	Water filtered (L)	DNA / cDNA yield (ng)	Raw read pairs (× 1000)	No. ASVs / ORFs
16S rRNA gene amplicons	QL-1679-1033	Before	MM-171.3	105	50.0	276	719
	QL-1679-1045	Before	MM-171.3	144	100	285	1,504
	QL-1679-1096	Before	MM-171.3	72.0	185	375	1,273
	QL-1679-1083	After	MM-171.3	64.0	640	250	1,321
	QL-1679-1085	After	MM-171.3	112	300	484	1,920
	QL-1679-1087	After	MM-171.3	143	55.0	667	1,010
	QL-1679-1077	Biofilm 20 d	MM-171.3		4.5	23.3	542
	QL-1679-1072	Biofilm 20 d	MM-171.3		< 0.05	37.5	168
	QL-1679-1071	Biofilm 40 d	MM-171.3		6.0	191	148
	QL-1679-1078	Biofilm 40 d	MM-171.3		10	6.04	51
	QL-1679-1073	Biofilm 75 d	MM-171.3		6.3	5.80	38
	QL-1679-1079	Biofilm 75 d	MM-171.3		7.5	10.3	57
	QL-1679-1023	Before	TM-448.4	385	50	224	508
	QL-1679-1035	Before	TM-448.4	338	15	277	537
	QL-1679-1059	Before	TM-448.4	172	20	63.3	505
	QL-1679-1089	After	TM-448.4	245	5.0	30.6	480
	QL-1679-1091	After	TM-448.4	161	2.5	9.21	379
	QL-1679-1093	After	TM-448.4	155	2.5	13.9	205
	QL-1679-1081	Biofilm 20 d	TM-448.4		< 0.05	8.31	133
	QL-1679-1082	Biofilm 20 d	TM-448.4		< 0.05	8.48	213
	QL-1679-1075	Biofilm 40 d	TM-448.4		11	224	52
	QL-1679-1074	Biofilm 40 d	TM-448.4		5.0	16.6	130
	QL-1679-1076	Biofilm 75 d	TM-448.4		4.5	5.57	36
	QL-1679-1080	Biofilm 75 d	TM-448.4		3.0	22.0	54
Meta-t	P10152_S2	Biofilm 75 d	MM-171.3		426	53.4 M	5,426
	P10152_S3	Biofilm 75 d	MM-171.3		463	32.4 M	5,194
	P10152_S1	Biofilm 75 d	TM-448.4		302	38.3 M	3,735
	P10152_S4	Biofilm 75 d	TM-448.4		466	44.2 M	4,251

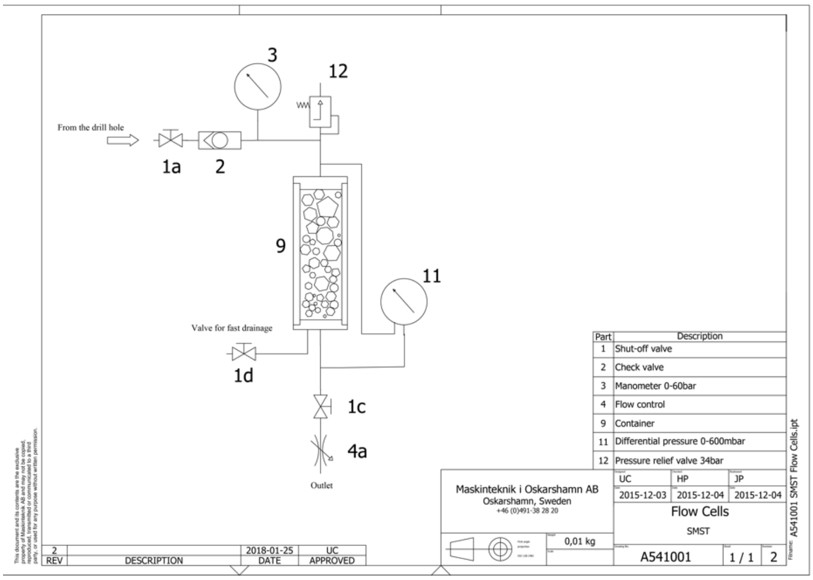

Supplementary Figure 1 Technical drawing of the flow-cell. Detailed drawing depicting the water flow from the borehole through the flow-cell.

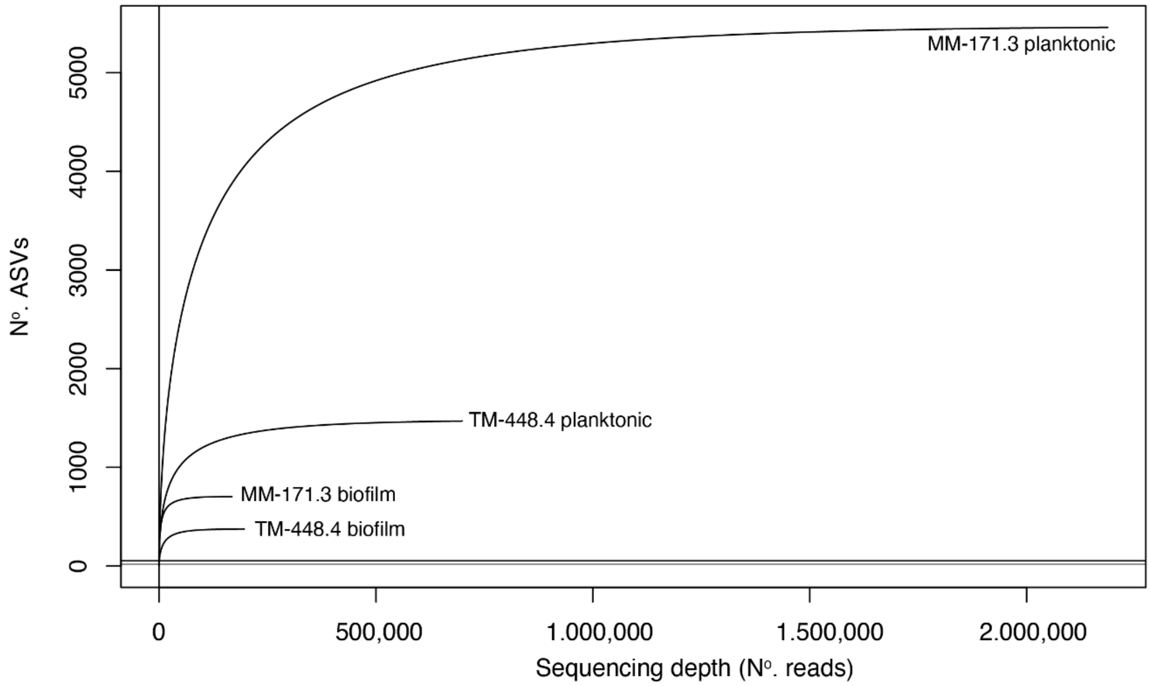

Supplementary Figure 2 Rarefaction curves for the 16S rRNA gene amplicons. The rarefied number of ASVs (y-axis) are plotted against the sequencing depth (number of reads, x-axis).

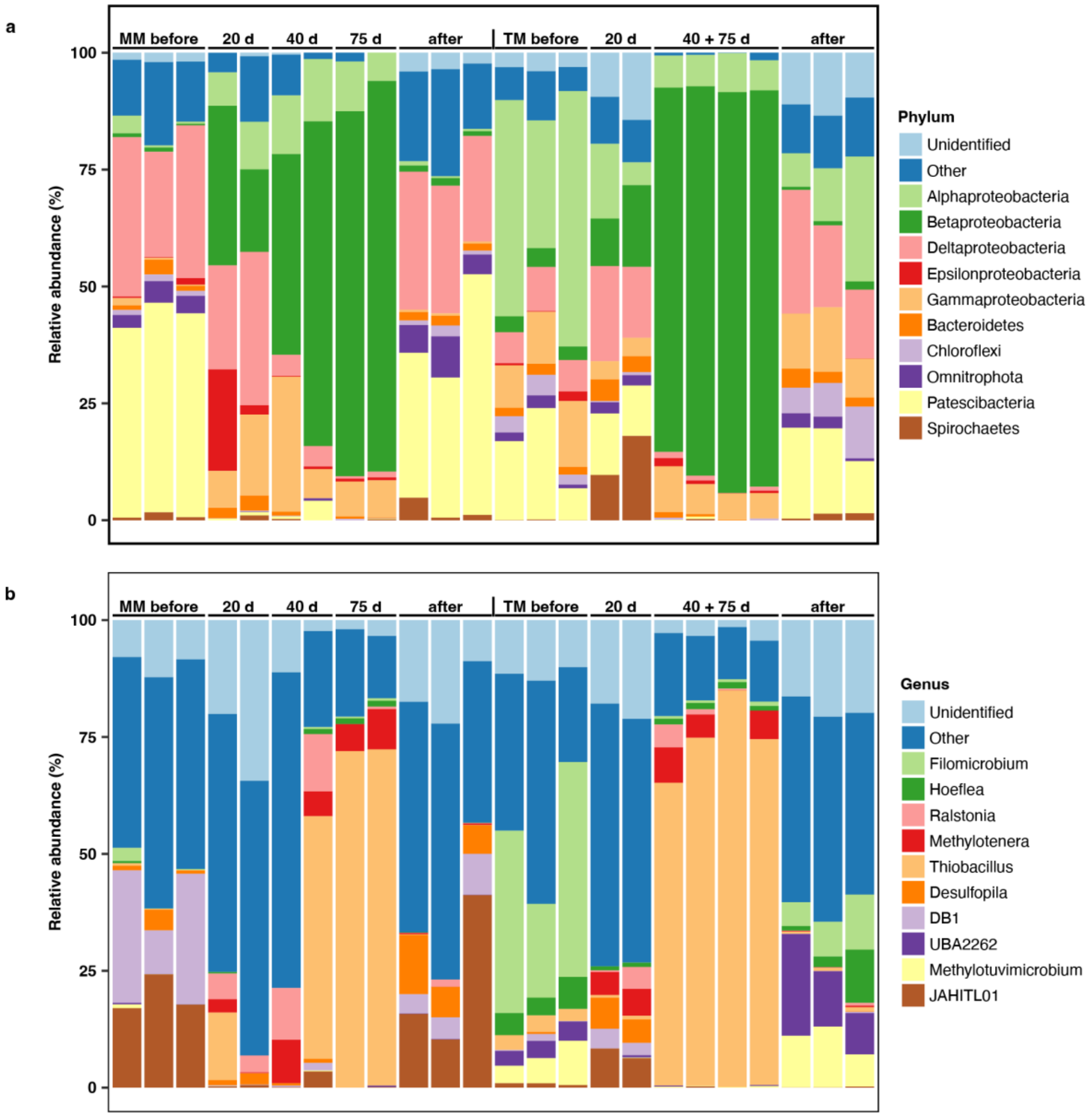

Supplementary Figure 3 Microbial community structure based on 16S rRNA gene amplicons. a) Community composition at the level of phylum for the planktonic and biofilm samples after 20, 40, and 75 days of biofilm development. The ten most abundant phyla are displayed with the Proteobacteria shown on the level of class and with the remaining taxa grouped as ‘Other'. ASVs not identified on the level of phylum grouped as "Unidentified". **b)** Community composition at the level of genus for the planktonic and biofilm samples after 20, 40, and 75 days of biofilm development. The ten most abundant genera are displayed with the remaining taxa grouped as ‘Other'. ASVs not identified on the level of genus grouped as "Unidentified".

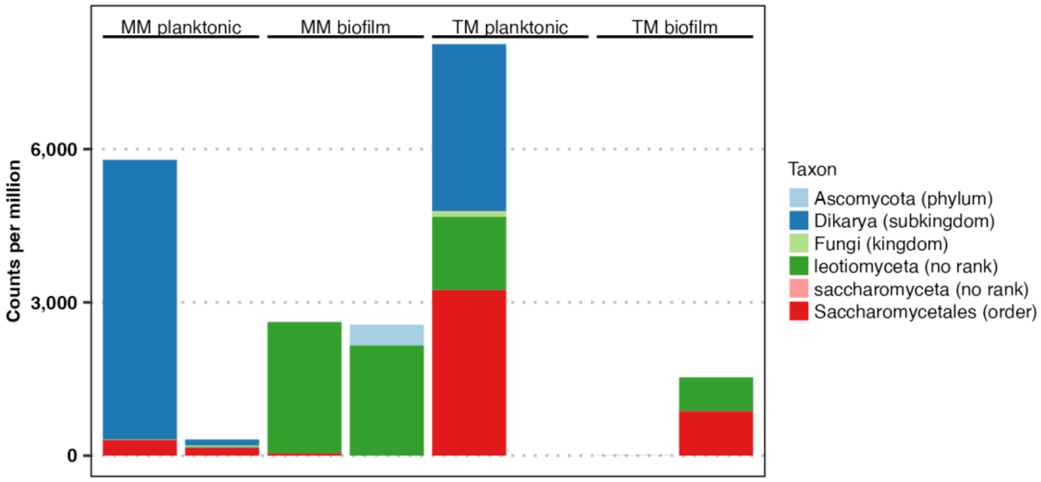

Supplementary Figure 4 Fungal RNA transcripts. The taxonomic groups are sorted at the lowest rank possible.

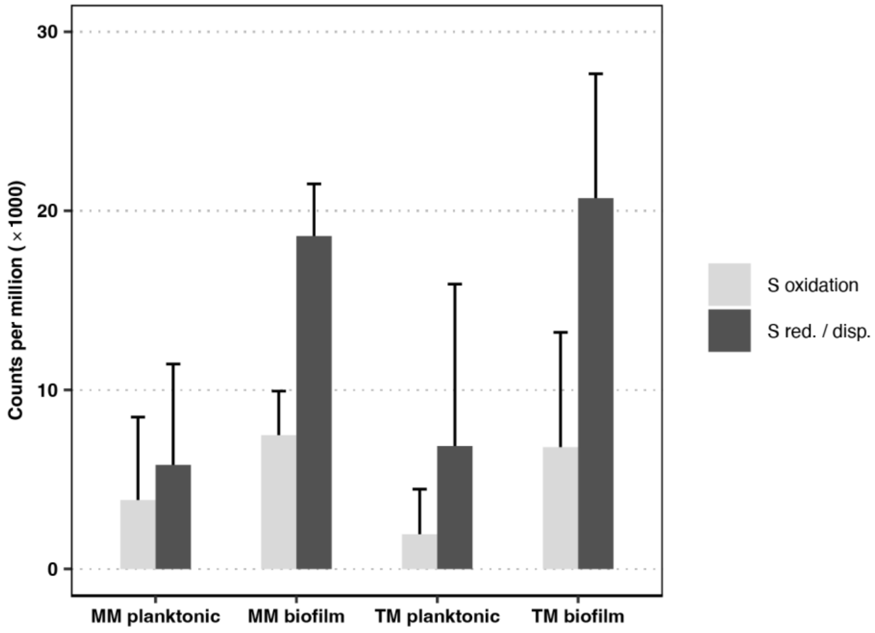

Supplementary Figure 5 Transcripts involved in sulfur metabolism. The category sulfur reduction and disproportionation comprised the genes sulfate adenylyltransferase (*sat*), dissimilatory sulfite reductase (*dsvB*C), adenylylsulfate reductase (*aprAB)*, anaerobic sulfite reductase (*asrAB*), and thiosulfate reductase (*phsAC*). The category sulfur oxidation comprised the genes thiosulfate oxidation (*soxA*), sulfite dehydrogenase (*soeABC* and *sorAB*), sulfide dehydrogenase (*fccB*), and sulfide oxidation (*sqr*). Error bars denote the standard deviation among the replicates (n = 2).

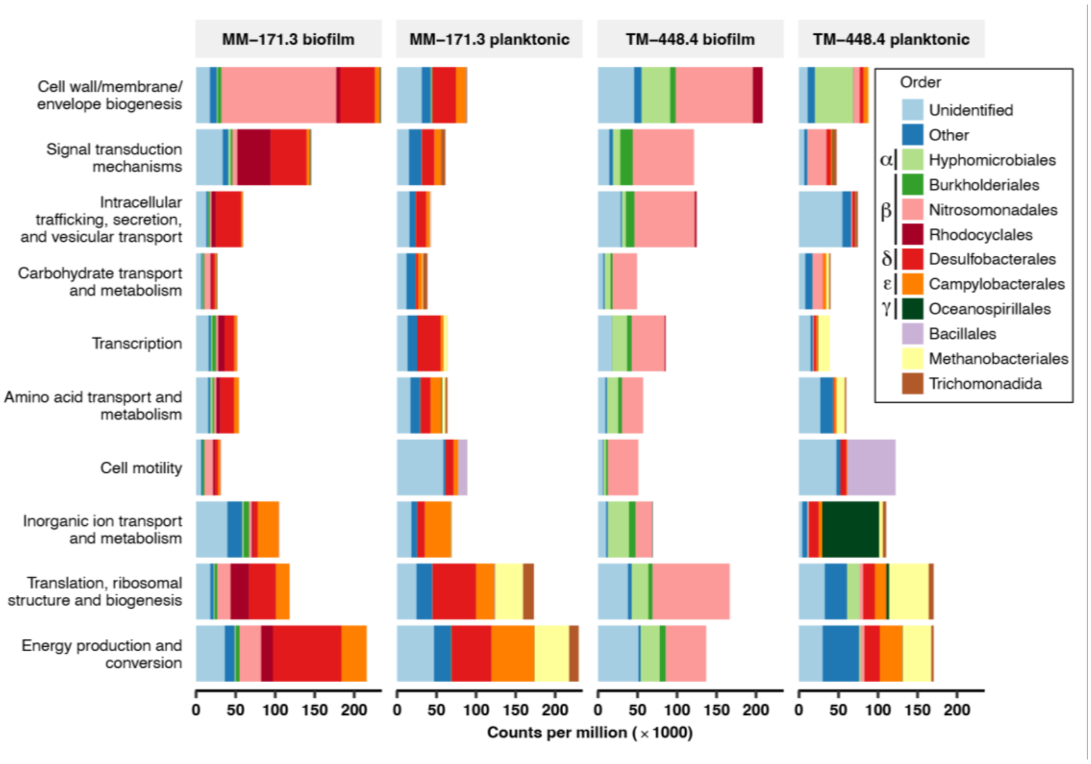

Supplementary Figure 6 Transcripts grouped according to functional category. Showing the abundance (in counts per million) of each COG category and the taxonomy (level of order) of the RNA transcripts comprising the functional groups. The ten most abundant orders are shown with the remaining taxa grouped as "Other" and transcripts not annotated on the level of order grouped as "Unidentified". Greek letters before the colour key denotes the class within the Proteobacteria (Alpha, Beta, Delta, and Epsilonproteobacteria)
